# The Genetic Underpinnings of Affective Temperaments: Identifying Novel Risk Variants with a Whole Genome Analytical Approach

**DOI:** 10.1192/j.eurpsy.2022.72

**Published:** 2022-09-01

**Authors:** X. Gonda, N. Eszlari, D. Torok, Z. Gal, A. Millinghoffer, P. Antal, G. Juhasz, G. Bagdy

**Affiliations:** 1 Semmelweis University, Department Of Psychiatry And Psychotherapy, Budapest, Hungary; 2 NAP-2-SE New Antidepressant Target Research Group, Hungarian Brain Research Program, Semmelweis University, Budapest, Hungary; 3 Semmelweis University, Department Of Pharmacodynamics, Budapest, Hungary; 4 Budapest University of Technology and Economics, Department Of Measurement And Information Systems, Budapest, Hungary; 5 SE-NAP-2 Genetic Brain Imaging Migraine Research Group, Semmelweis University, Budapest, Hungary

**Keywords:** affective disorders, Genetics, GWAS, affective temperaments

## Abstract

One reason behind the failure to understand the neurobiological background of affective disorders and develop more effective treatments is their heterogeneity warranting identification of clinically meaningful endophenotypes. Affective temperaments, considered subclinical manifestations and pathoplastic contributors of affective illnesses may constitute such endophenotypes. 775 general population subjects were phenotyped for affective temperaments using TEMPS-A, and genotyped using Illumina’s CoreExom PsychChip yielding 573141 variants. Primary SNP-based association tests were calculated using linear regression models assuming an additive genetic effect with the first 10 calculated principal components, gender, age, and other affective temperaments as covariates; a Bonferroni-corrected genome-wide significance threshold set at p≤5.0×10^−8^, and suggestive significance threshold set at p≤1.0x10^-5^. SNP-level relevances were aggregated to gene-level with the PEGASUS method, with a Bonferroni-corrected significance level set at 2.0×10^-6^, and suggestive significance thrshold set at p≤4.0×10^-4^. Functional effects of most significant SNPs as reported in public open databases based on expression quantitative trait loci (eQTL) and 3D-chromatin interactions were explored using FUMA v1.3.5. In SNP-based tests a novel genome-wide significant variant, rs3798978 (p=4.44x10^-8^) and several other suggestively significant SNPs in ADGRB3 were found for anxious temperament along with suggestively significant SNPs for the other four affective temperaments. In gene-based tests suggestively significant findings emerged for all five temperaments. Functional analysis suggested that several of the identified variants influence gene-expression levels or participate in chromatin interactions in brain areas implicated in affective disorders. In the next step these findings should be investigated in patient samples, and in other models of affective disorders and related phenotypes.

**Disclosure:**

No significant relationships.

